# Th17 Cells Coordinate with Th22 Cells in Maintaining Homeostasis of Intestinal Tissues and both are Depleted in SIV-Infected Macaques

**DOI:** 10.4172/2155-6113.1000302

**Published:** 2014-05

**Authors:** Huanbin Xu, Xiaolei Wang, Ronald S. Veazey

**Affiliations:** Tulane National Primate Research Center, Tulane University School of Medicine, 18703 Three Rivers Road Covington, LA 70433, USA

**Keywords:** Th17, Th22, SIV, HIV, Intestine, Mucosal immunology

## Abstract

Th17 and Th22 cells are thought to function as innate regulators of mucosal antimicrobial responses, tissue inflammation and mucosal integrity, yet their role in persistent SIV infection is still unclear. Here we compared Th17 and Th22 cells in their phenotype, effector/cytokine function, and frequency in blood and intestinal mucosal tissues, and correlate levels with mucosal damage in SIV-infected rhesus macaques. We found that Th17/Th22 cells share similar features in that both highly produce TNF-α and IL-2 and express CCR5 in intestinal tissues; yet very few show cytotoxic functions, as evidenced by lack of IFN-γ and granzyme B production. Further, Th17/Th22 cells display distinct tissue-specific distributions. Both Th17 and Th22 cells and cytokine secretion were significantly depleted in both blood and intestine in chronically SIV-infected macaques. The frequency of Th17 and Th22 cells in the intestine positively correlated with percentages of intestinal CD4+ T cells and negatively with damage to intestinal mucosa, and plasma viral loads in SIV infection. These findings indicate Th17 and Th22 cells share considerable functions, and may coordinate in innate mucosal immune responses, and their regional loss in the intestine may be associated with local mucosal immune dysfunction in persistent HIV/SIV infection.

## Introduction

Progressive human immunodeficiency virus (HIV) and simian immunodeficiency virus (SIV) infection is characterized by massive, early loss of CD4 T cells from the intestinal mucosa, as well as by structural disruption of the gut barrier and increased microbial translocation [1–4]. We and others have shown IL-17-producing cells are preferentially depleted in the gastrointestinal (GI) tracts of HIV-infected humans and SIV-infected rhesus macaques (RMs) [5–7]. In contrast, Th17 cells are maintained at relatively normal levels in the GI tract of SIV-infected natural host species, HIV-infected long-term nonprogressors, as well as some patients on antiviral therapy [8–11]. Since they are mostly restricted to mucosal tissues, and since SIV and HIV largely replicate in intestinal tissues, it has been suggested these cells may help control infection, as indicated by experiments in macaques correlating levels of the pre-existing Th17 cell pool with subsequent limited SIV replication [12,13]. However, selective loss of Th17 in mucosal tissues is associated with compromise of the mucosal barrier, resulting in dissemination of microbial products through the gut into the systemic circulation, [14], which in turn results in systemic immune activation, thus contributing to SIV/HIV pathogenesis [3,12].

Th17 cells are able to produce effector cytokines including IL-17, IL-21 and IL-22, and mediate host defense against a variety of intestinal pathogens, and they have also been implicated in the pathogenesis of autoimmune diseases [15–20]. In addition to host defense and inflammation, IL-17 is also crucial for maintenance of tight junctions between intestinal epithelial cells [21]. Notably, IL-22 is made by both Th17 cells and innate lymphoid cells (ILC), as well as a distinct subset of CD4 helper T cells termed Th22 cells, which are an important source of IL-22 [6,22]. IL-22 is important in promoting innate immune defenses against bacterial and fungal infections in mucosal tissues, and in maintaining mucosal barrier integrity, mucus production, and mucosal tissue remodeling and repair [23–29]. Although Th17 and Th22 cells share some features, they apparently have distinct roles in the pathogenesis of diseases [30]. Similar to Th17 cells, Th22 cells represent a distinct subset of T helper cells, with differentiation-dependent transcription factors distinct from those of Th1, Th2, and Th17 cells. These cells produce IL-22 independent of IFN-γ and IL-17 [31–33] and their genes encode proteins involved in tissue remodeling and angiogenesis [32]. Th22 cells are also dramatically depleted during chronic HIV infection, accompanied by compromised epithelial integrity and increased microbial translocation [29,34]. Th17/Th22 cells may also respond differently to different microenvironments or diseases such as infection, autoimmune disease or allergy [35,36]. However, the characterization, and dynamics of Th17/Th22 cells in HIV/SIV infection is still unclear.

Here we characterized the phenotype, polyclonal functions, and tissue distribution of Th17 and Th22 cells in peripheral blood and intestinal tissues, examined the effects of SIV infection on Th17/Th22 subsets in SIV-infected macaques, and correlated their levels with intestinal CD4 T cells, disruption of intestinal mucosal tissues *in vivo*, and maintenance of epithelial cells *in vitro*. Our data show that both IL-17 and IL-22-producing (Th17/Th22) cells are significantly depleted in blood and intestine in persistently SIV-infected animals, especially in intestinal tissues, in which Th17/Th22 cells highly express CCR5. Loss of intestinal Th17 and Th22 cells correlated with reductions in intestinal CD4+ T cells during SIV infection, accompanied by damage to the mucosal barrier. Moreover, we show both IL-17 and IL-22 promote intestinal epithelial survival *in vitro*. These findings suggest that Th17 cells functionally cooperate with Th22 cells, and both appear critical in maintaining innate immunity and regulating homeostasis of the gut barrier in HIV/SIV infection.

## Results

### Characterization of Th17 and Th22 cells in healthy rhesus macaques

**Figure 1:**
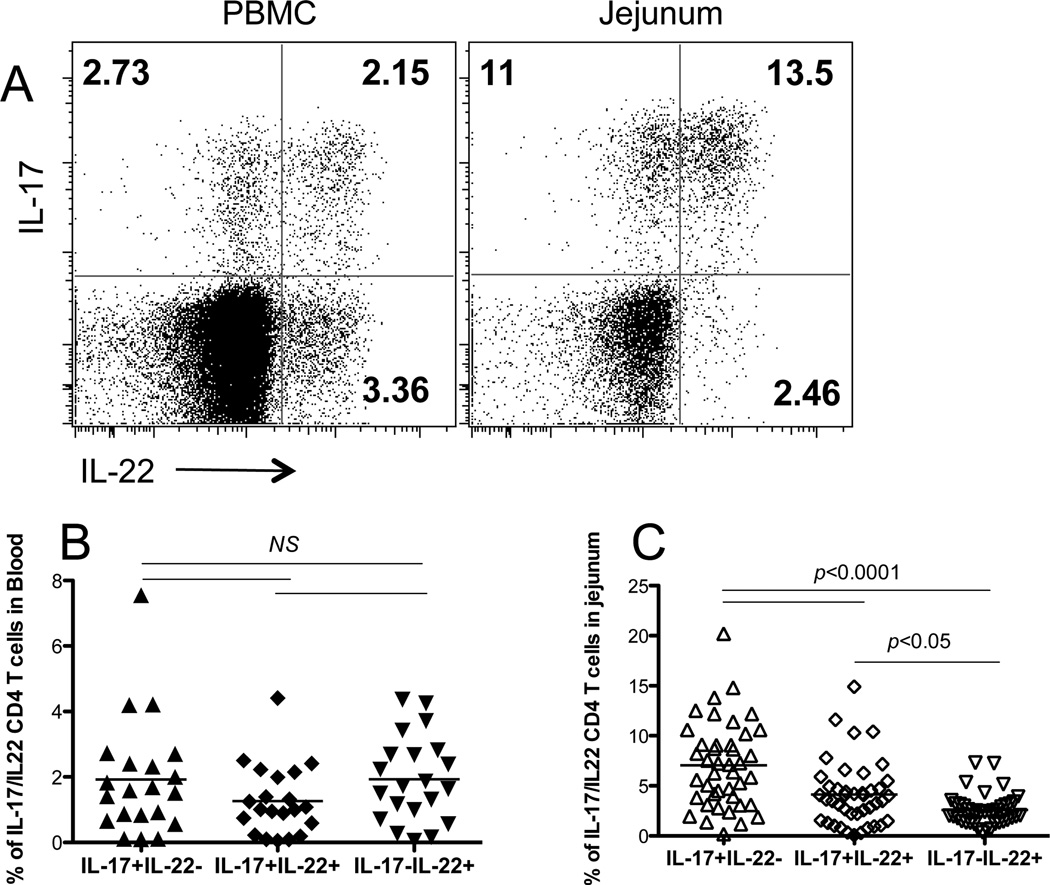
Characterization of IL-17 and IL-22-producing CD4 T cells in peripheral blood and jejunum of uninfected macaques (A) Representative dot plots of IL-17 and/or IL-22-producing cells gated through CD3+CD4+ T cells in peripheral blood and jejunum. (B) Frequency and tissue specificity of Th17/Th22 cell subsets: percentages of IL-17+IL-22-, IL-17+IL-22+ and IL-17-IL-22+ cells in peripheral blood (n=21) and jejunum (n=42) after gating through CD3+CD4+ lymphocytes. Note the majority of IL-22+ cells from the intestine also produce IL-17, but not those from blood.

Th17 and Th22 cells were defined here defined based on CD3+CD4+ expression, and their ability to produce IL-17 or/and IL-22, and levels of each subset were compared in peripheral blood and intestinal tissues of normal (uninfected) macaques. As shown in Figure 1A and 1B, Th17 cells were present in peripheral blood and intestinal mucosal tissues, yet Th17 cells could be divided into two populations based on IL-22 secretion. Similarly, some Th22 cells also produced IL-17, suggesting Th17/Th22 cells share overlapping functions. However, Th22 cells showed tissue-specific distribution, as considerable numbers of IL-17+ (2.15%) or IL-17-Th22 cells (3.36%) were detected in peripheral blood, yet dual positive IL-17+IL-22+ Th22 cells were predominantly found in intestinal tissues (13.5% vs 2.46%). Notably, the Th22 cell subsets, which produced IL-22 but not IL-17, were relatively rare in mucosal tissues (Figure 1C). As shown in Figure 1B and 1C, frequencies of IL-17+IL-22-, IL-17+IL-22+ and IL-17-IL-22+ CD4 T cell subsets were similar in blood, however, only a small fraction of IL-17-IL-22+ CD4 T cells were detected in intestinal tissues (Figure 1).

**Figure 2:**
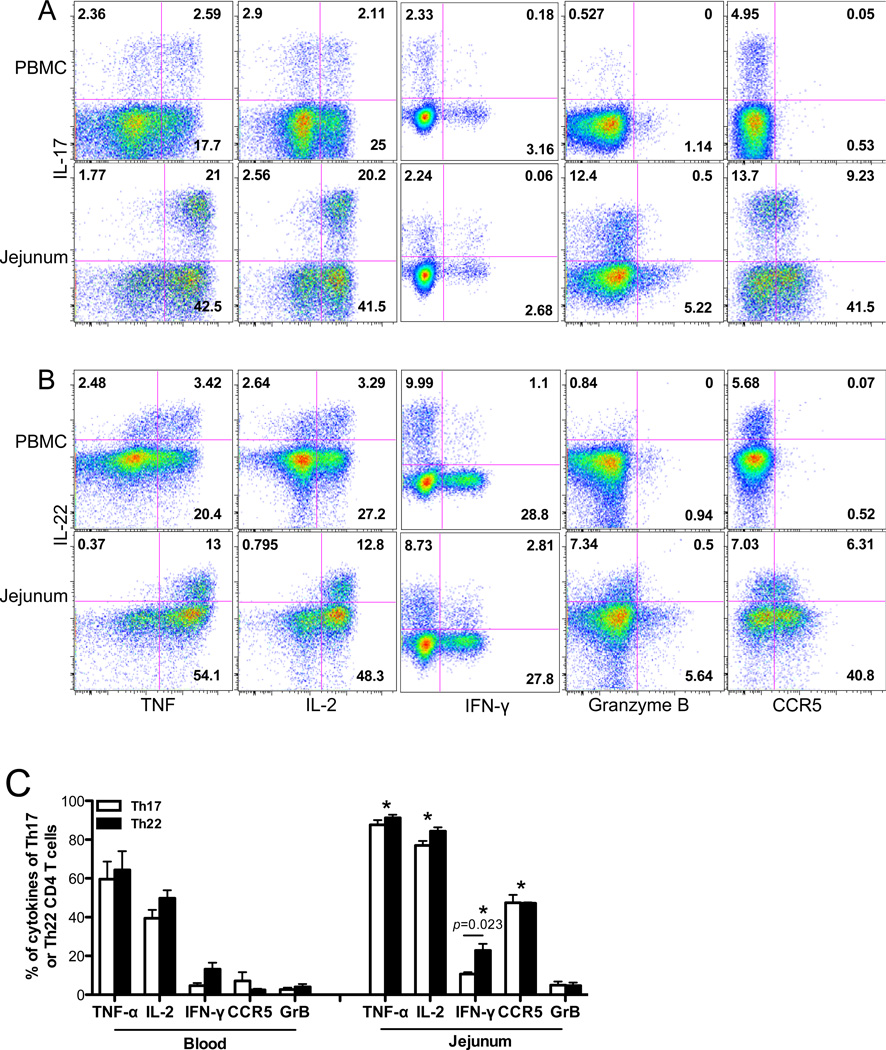
Production of effector cytokines and CCR5 expression by Th17 and Th22 cells from blood and intestinal tissues of macaques Dot plots showing TNF-α, IL-2, IFN-γ, Granzyme B and CCR5 expression of IL-17+ (A) or IL-22+ CD4 T cells (B) from blood and jejunum in uninfected macaques; (C) statistical comparison of cytokine-producing capability of Th17 and Th22 cells in peripheral blood and jejunum. * p<0.05, compared with peripheral blood-derived cells.

We further examined the ability of Th17/Th22 cells isolated from peripheral and intestinal mucosal tissues to produce other effector cytokines. Approximately 50% of peripheral blood Th17 cells were able to secret TNF-α or IL-2. In contrast, almost all Th17 cells from intestinal tissues produced TNF-α and IL-2 (Figure 2A, *p*<0.05). Both peripheral and intestinal Th17 cells produced little IFN-γ and undetectable levels of granzyme B, suggesting these were not cytotoxic cells. Moreover, blood Th17 cells did not express the major HIV/SIV coreceptor CCR5, yet considerable numbers of intestinal Th17 cells (~40%) were CCR5-positive (Figure 2A), consistent with previous reports [8]. However, in comparison to Th17 cells, Th22 cells displayed very similar tissue-specific patterns of TNF-α and IL-2 secretion, low levels of granzyme B and high CCR5 expression (Figure 2B). Notably, intestinal Th22 cells were distinct from Th17 cells, as indicated by slightly higher capacity to produce IFN-γ compared to either peripheral or intestinal Th17 cells (Figure 2C). These data suggest that Th17/Th22 cells share many innate immune functions, and many are direct viral target cells for HIV/SIV infection, at least in the intestinal tract. Nonetheless, some intestinal and blood Th17 and Th22 cells represent distinct functional cell populations as evidenced by the existence of separate IL-22-Th17 and IL-17-Th22 cell subsets in tissues (Figure 2). Collectively, these common and divergent features suggest that Th17 cells and Th22 cells have many overlapping functions, and both IL-17 and IL-22 may play cooperative roles in regulating mucosal immune responses.

### Depletion of both Th17 and Th22 cell subsets occurs in SIV-infected macaques

**Figure 3:**
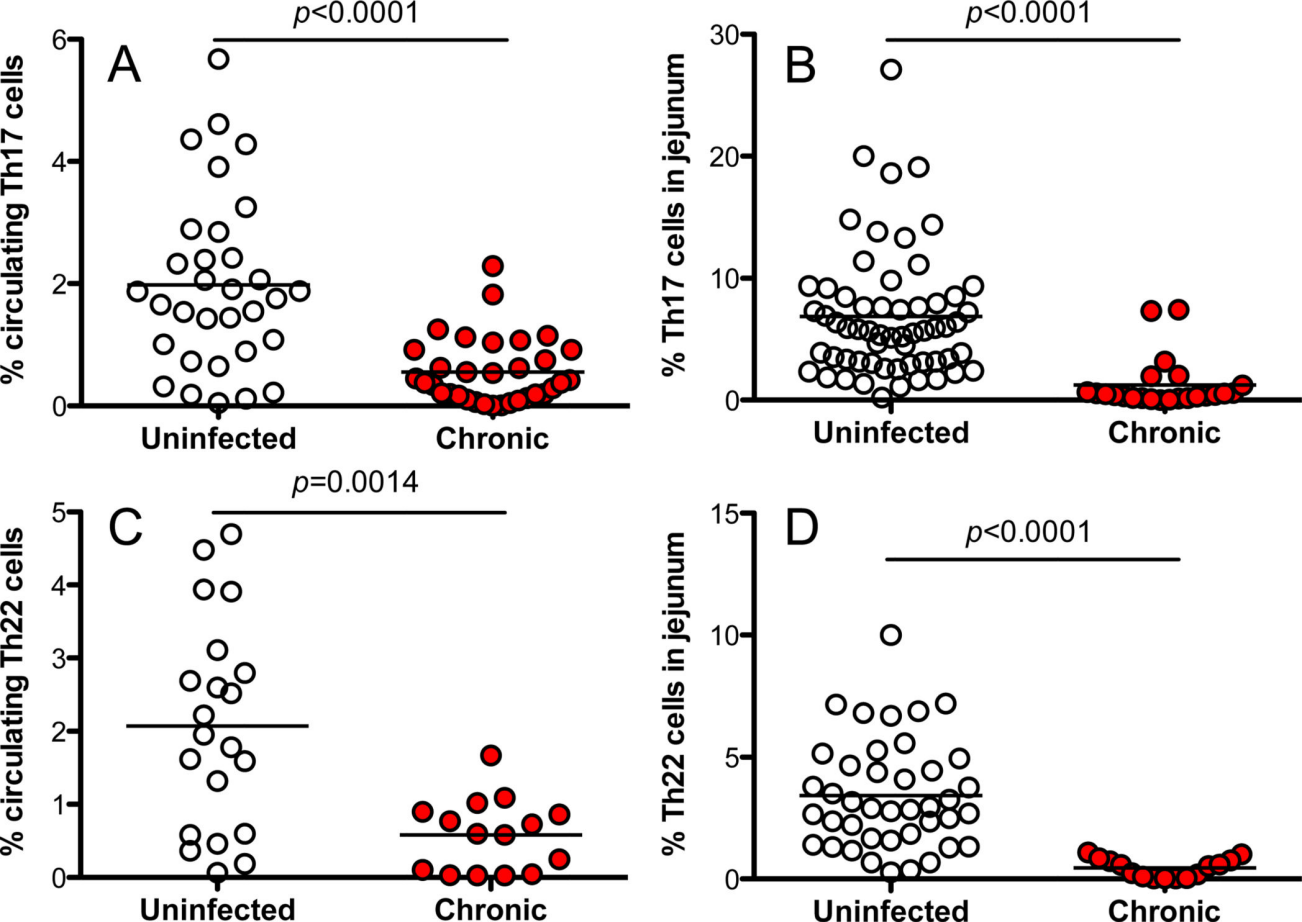
Depletion of blood and intestinal Th17 and Th22 CD4 T cells in chronic SIV infection of macaques Comparison of percentages of; (A) circulating IL-17+Th17 cells between uninfected (n=31) and chronically SIV-infected (n=33) rhesus macaques (RM); (B) intestinal IL-17+Th17 cells between uninfected (n=60) and chronically SIV-infected (n=23) RM; C) circulating IL-22+Th22 cells between uninfected (n=21) and chronically SIV-infected (n=15) RM, and; (D) intestinal IL-22+Th22 cells between uninfected (n=41) and chronically SIV-infected (n=15) RM. Th17/Th22 cells were generated by first gated through CD3+CD4+ T cells and identified by

**Figure 4:**
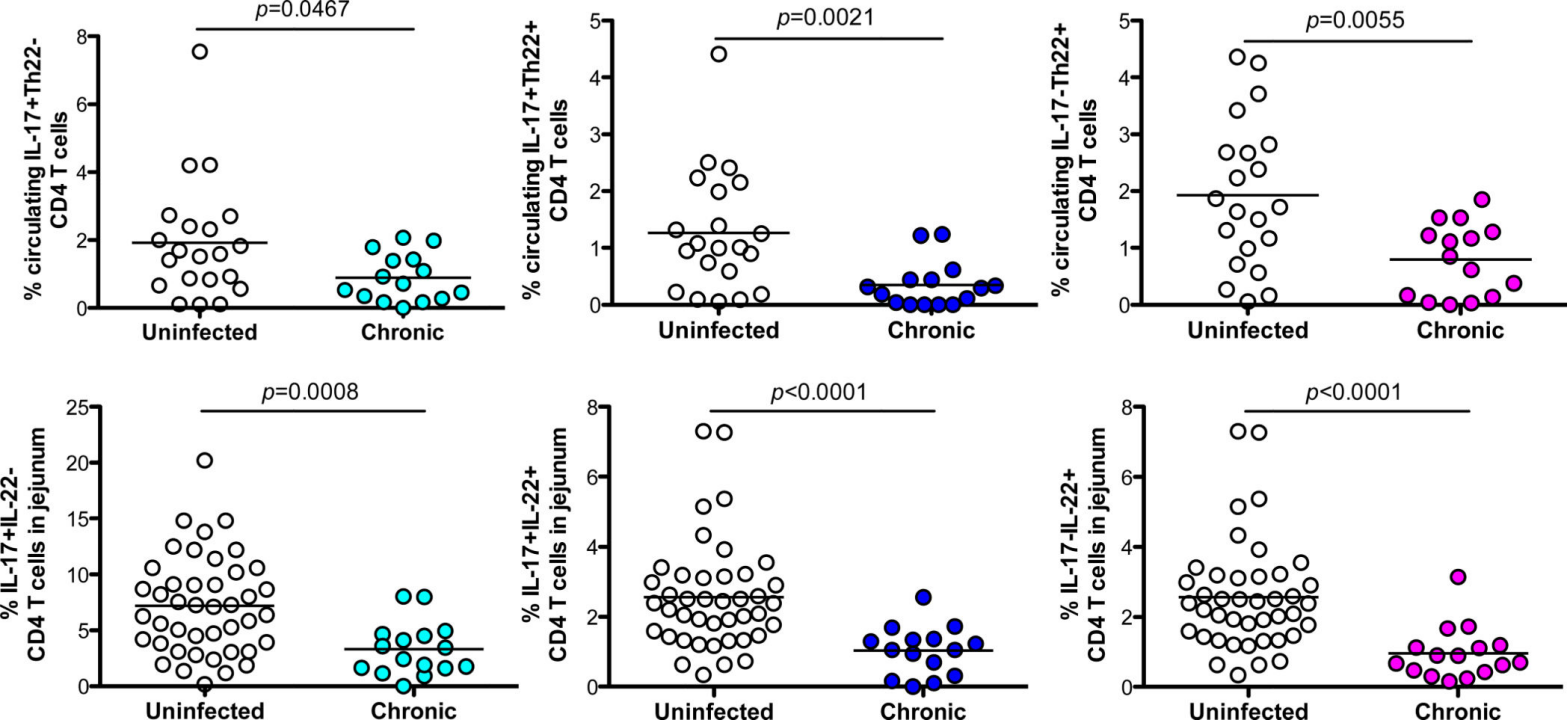
Comparison of blood and intestinal IL-17+IL-22-, IL-17+IL-22+ or IL-17-IL-22+ CD4 T cell subsets in chronic SIV infection of macaques Percentages of IL-17+IL-22-, IL-17+IL-22+ or IL-17-IL-22+ cell subsets in peripheral blood and intestinal mucosal tissues in uninfected (Blood, n=21; Jejunum, n=42) and chronically SIV-infected (Blood, n=15; Jejunum, n=16) macaques are shown. Statistical analyses show significant differences in levels of IL-17+IL-22-, IL-17+IL-22+ or IL-17-IL-22+ cells in blood and intestine between SIV-infected and uninfected animals.

To investigate the effects of persistent SIV infection on Th17/Th22 cell subsets in rhesus macaques, we examined their changes in peripheral blood and jejunum. In normal, healthy animals, the frequency of Th17 and Th22 cells were similar in blood (1.98% vs. 2.07%, respectively) (Figure 3A and 3C), but levels of Th17 cells were significantly higher than Th22 cells in jejunum (6.85% vs. 3.43%, *p*<0.0001) (Figure 3B and 3D). There was also a substantial reduction in frequency of both Th17 and Th22 cells in the peripheral blood and intestinal mucosal tissues in chronically SIV-infected animals, compared with uninfected controls (p<0.001) (Figure 3). As shown in Figure 4, all Th17/Th22 cell subsets in blood and mucosal tissues were significantly depleted in persistent SIV infection. Thus, despite distinct differences in cytokine production, both subsets are depleted, resulting in loss of the major sources of both IL-17 and IL-22 in tissues, which likely contributes to the pathogenesis of HIV/SIV infection.

### Th17 and Th22 cells are involved in homeostasis of intestinal epithelial cells, and their loss correlates with disruption of mucosal lymphoid tissues and intestinal CD4+ T cell depletion

**Figure 5:**
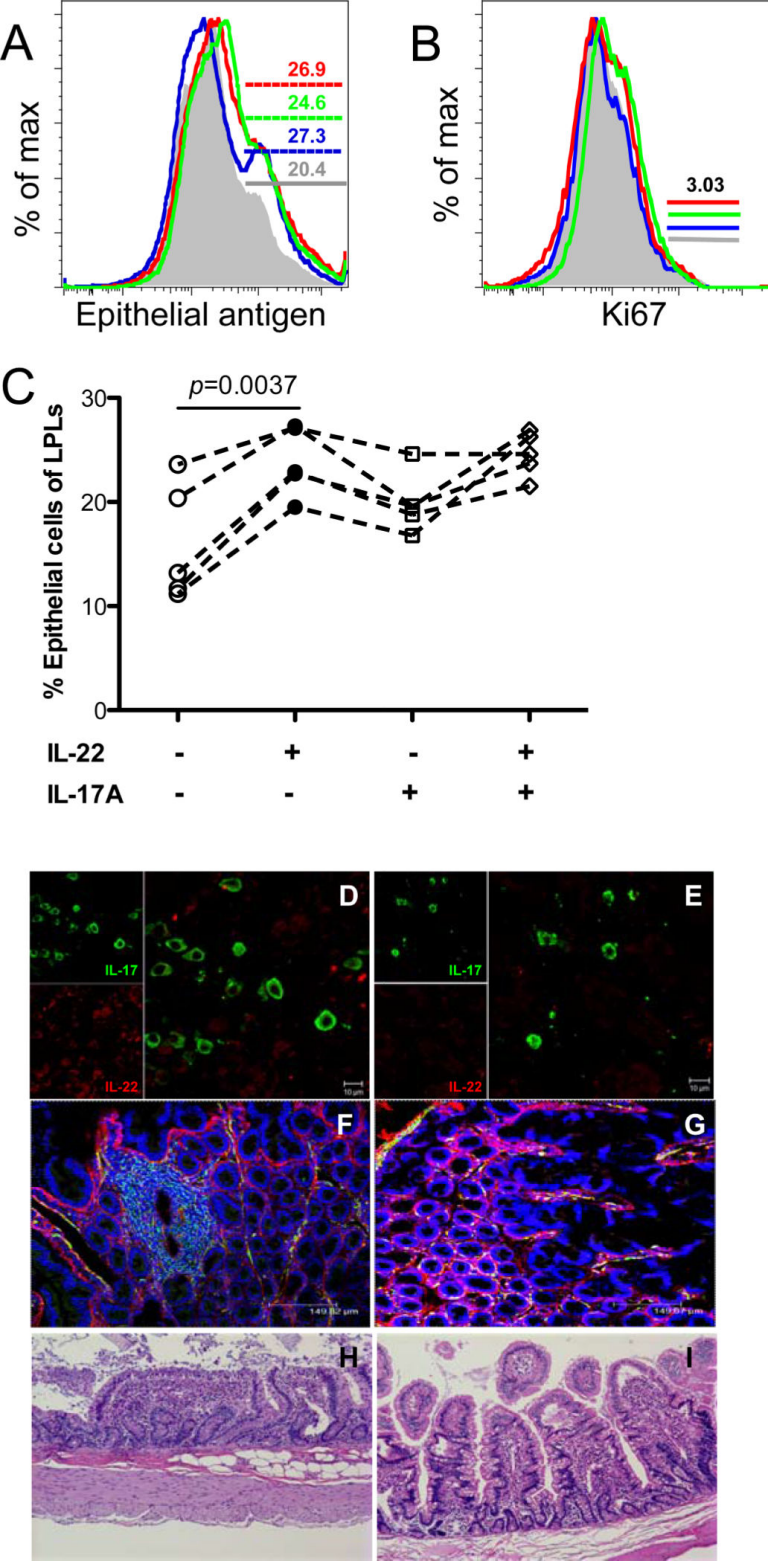
Effects of IL-17A/IL-22 on intestinal epithelial cell survival *in vitro* and correlation of IL-17+ and IL-22+ cells with mucosal pathology in intestinal tissues of SIV-infected macaques *in situ* Representative histogram of intestinal epithelial cells (A) and their proliferation (B) after in vitro stimulation with IL-22 (blue), IL-17A (green), or both (red) compared with media controls (grey) after 24h culture; (C) Percentage of live epithelial cells after stimulation with IL-22, IL-17A, or both compared to media alone controls after 24h culture. The data in (C) show results from five different normal (uninfected) animals. (D and E) Confocal microscopy showing IL-17 (green) and IL-22 (red)-producing cells in mitogen-stimulated intestinal explants from uninfected (D) and chronically SIV-infected (E) macaques. Scale bar, 10µm. Note the loss of IL-22+ cells in SIV-infected intestinal tissues. (F and G) Confocal microscopy showing desmin (green), collagen (red) and nuclei (blue) in jejunum from a chronically SIV-infected (F) and uninfected (G) macaque. Note increased collagen deposition in the lamina propria of the infected macaque; (H and I) Hematoxylin and Eosin stained sections of jejunum from a chronically SIV-infected macaque (H) and uninfected macaque (I) (10×). Note intestinal villus blunting and fusion in the jejunum of the SIV infected macaque.

Since both IL-17 and IL-22 play important roles in maintaining mucosal barrier integrity and regulating the function of epithelial cells [21,26,37], we stimulated intestinal cells with IL-22 or/and IL-17A for 24h immediately *ex vivo*, and compared the frequency of live epithelial cells after 24 hrs culture. Percentages of epithelial cells were consistently higher in the presence of IL-22, IL-17A or both, compared with media controls without stimulation (Figure 3A and 3C). However, there were no significant differences in rates of epithelial cell proliferation (Ki-67 expression) between stimulated cells and controls under transient culture (24hrs) (Figure 3B), Nonetheless, increased survival of epithelial cells ex vivo suggest that IL-22 and IL-17A are both critical for maintaining the viability and homeostasis of intestinal epithelial cells. We further examined IL-17 and IL-22 producing cells in intestinal tissues by confocal microscopy. In uninfected animals, single-stained IL-17+, IL-22+ or dual-stained IL-17+IL-22+ cells were examined in the intestine (Figure 5D). Notably, both IL-17 and IL-22-producing cells were markedly reduced in chronically SIV-infected macaques (Figure 5E), consistent with the flow cytometry data (Figures 3 and 4). Both immunohistochemistry and histopathology showed SIV-infected animals had mucosal damage characterized by increased collagen deposition (Figure 5F and G), and blunting, shortening, and fusion of intestinal villi in the intestinal lamina propria (Figure 5F and 5H), compared with uninfected controls (Figure 5G and 5I). These findings suggest that loss of Th17/Th22 cells, and the local loss of their crucial cytokines in intestinal tissues is linked with compromised homeostasis of the intestinal mucosal barrier, resulting in the microbial translocation and systemic activation characteristic of SIV and HIV infections.

**Figure 6:**
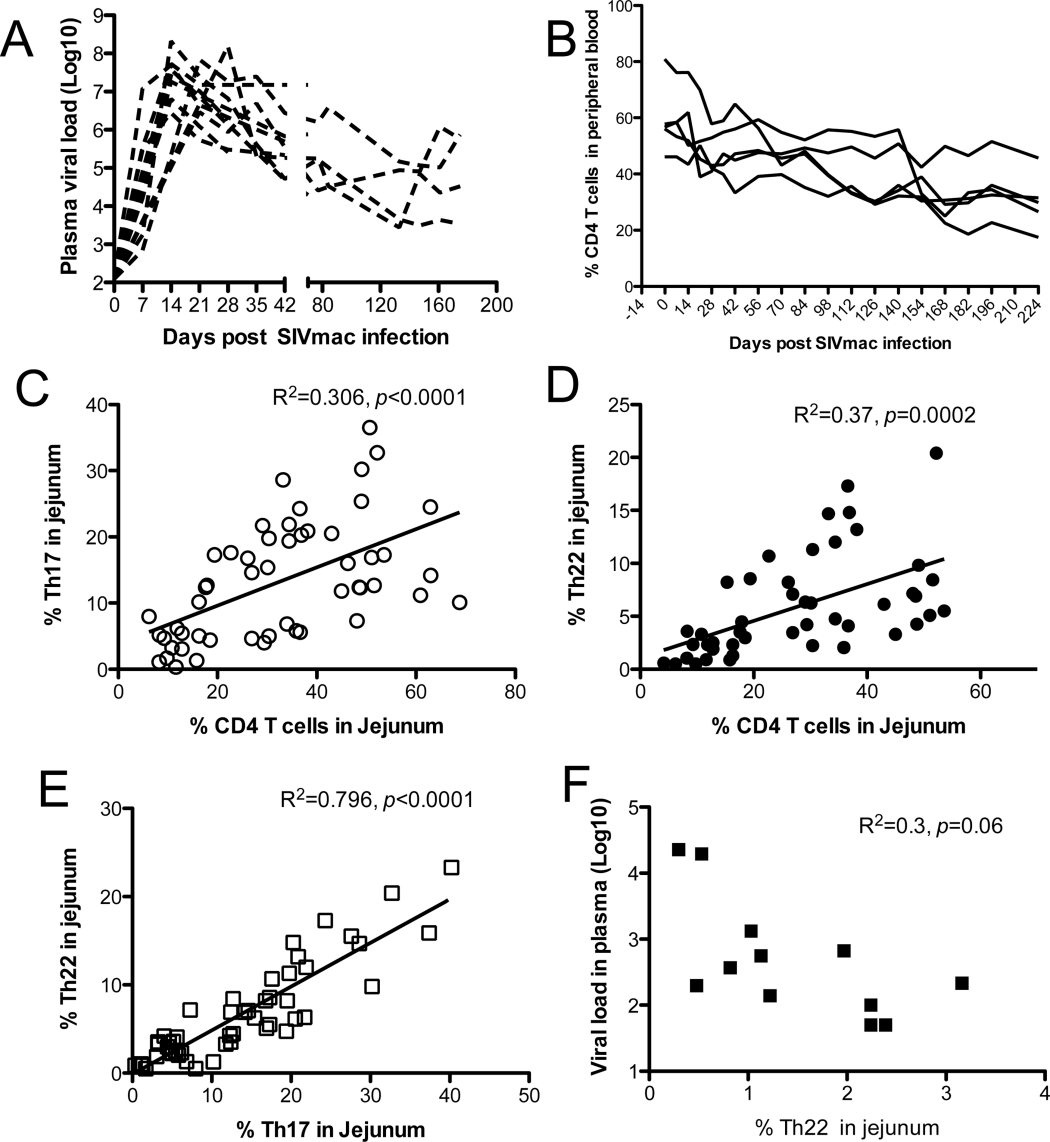
Loss of intestinal Th17 or Th22 cells correlates with preferential depletion of intestinal CD4 T cells during SIV infection in macaques (A) Viral load in plasma in macaques post SIV infection (n=11). (B) Longitudinal examination of circulating CD4 T cells in macaques from primary to chronic SIV infection (n=5). (C and D) Positive correlations of Th17 or Th22 cells with total CD4 T cells in intestinal mucosal tissues. (E) Positive correlations between Th17 and Th22 cells in intestinal mucosal tissues. Uninfected, n=41; chronic SIV, n=15. (F) Inverse correlation of intestinal Th22 cells with plasma viral loads in chronic SIV infection (n=12).

Levels of SIV peak in plasma 14~21 days after infection of macaques, followed by gradual reductions in peripheral CD4 T cells (Figure 6A and 6B). However, HIV/SIV infection is characterized by profound and rapid depletion of CD4 T cells in the gastrointestinal tract, and levels of residual CD4 T cells inversely correlate with maintenance of intestinal architecture and disease progression in AIDS [38–40]. Increasing evidence suggests specific subsets of CD4+ T cells (Th17/Th22) are responsible for maintaining mucosal epithelial integrity. We thus correlated levels of Th17/Th22 cells with total intestinal CD4 T cells in the GI tract, and with plasma viral loads during SIV infection. The results showed that levels of intestinal Th17 cells positively correlated with both total CD4 T cells (R^2^=0.306, p<0.0001) (Figure 6C) and Th22 cells (R^2^=0.796, p<0.0001) (Figure 6E) during SIV infection. Similarly, levels of Th22 cells also positively correlated with total CD4 T cells in intestine, and indirectly with plasma viral loads in plasma in SIV infection (Figure 6D and F), consistent with previous reports describing disease progression associated with the loss of Th17 and/or Th22 cells [7,41].

## Discussion

HIV/SIV infection results in massive loss of CD4 T cells in GI tract, accompanied by compromised mucosal immunity, disruption of the mucosal barrier, and subsequent microbial translocation associated with sustained systemic immune activation that drives HIV replication and persistence. Here, we simultaneously characterized distinct subsets of Th17 and Th22 CD4 T cells in peripheral blood and in the small intestine (jejunum), compared their levels in chronically SIV-infected macaques with uninfected controls, and correlated their levels with disruption of intestinal mucosal architecture. Similar to prior reports in the large intestine [7] these results show that IL-17 and IL-22-producing (Th17/Th22) cells share common features and functions including their distribution, phenotype, and cytokine production, yet both are largely restricted to mucosal tissues. Both subsets were persistently depleted in intestines of SIV-infected animals, and their depletion directly correlates with loss of total intestinal CD4+ T cells during SIV infection. These findings suggest that Th17 and Th22 cells have overlapping functions, yet are both critical in maintaining innate immunity and mucosal integrity.

Th17 and Th22 cells represent distinct CD4 T cell lineages, yet they possess a number of similar features [30–33]. Th17/Th22 cells are involved in host defense, maintenance of mucosal integrity and have been implicated in the pathogenesis of autoimmune disease [13,23–25,42,43]. Th17 cell differentiation is controlled by the transcription factor retinoid orphan receptor γt (RORγt), and these cells are often able to also produce IL-22 [44], thus sharing some functions with Th22 cells. Th22 cells are mostly localized to mucosal tissues, and are crucial for protecting host mucosal tissues against bacterial infections by their IL-22 production [45]. Further, although innate lymphoid cells (ILCs) are also able to produce IL-22 [6], Th22 cells are considered to be a major source of IL-22 [22]. Here we found that IL-17+IL-22+ CD4 T cells were the predominant Th22 cell type in intestinal tissues, with far higher frequencies than IL-17-Th22 cells (Figure 1A and 1C). Based on their relative proportions and capacity for secretion in tissues and blood, we speculate that IL-17+IL-22+ CD4 T cells may be Th22 cells, but not Th17 cells, but these relationships need to be further investigated. Nonetheless, it is clear that Th17 and Th22 cells have overlapping functions, and may play coordinated roles in maintaining innate immunity and mucosal structure.

SIV infection resulted in marked reductions in both Th17/Th22 cells, especially in the jejunum, in which mucosal lymphoid tissues were disrupted. Similar results were recently reported in the large intestine of SIV-infected macaques [7]. We speculate this loss is due to direct viral infection since at least in the gut, these cells express both CD4+ and CCR5+. Circulating blood Th22 cells express higher levels of the HIV co-receptor CCR5 than Th17 cells in humans [34]. However, expression of CCR5 appears tissue-specific for both Th17 and Th22 cells, as both are mostly CCR5-negative in the peripheral blood yet highly positive for CCR5 in the gastrointestinal tract. This is also consistent with other reports of CCR5 expression on Th17 cells in RMs [8,46] which suggests intestinal Th17/Th22 cells could be direct targets of HIV/SIV. In macaques with chronic SIV infection, very few IL17/IL-22-producing cells were detectable (Figurescells in elite control over HIV 3–5). This loss of Th17/Th22 cells, which are a major source of IL17/IL22 in the gut, correlated with reductions of total intestinal CD4+ T cells (Figure 6), and combined, may be a major factor attributing to the breakdown of mucosal epithelial integrity [7,47], leading to microbial translocation and chronic immune activation [3]. It is increasingly recognized that IL-17, in combination with IL-22, is crucial for protection against bacterial infections in mucosal tissues, and for maintenance of the mucosal barrier by promoting intestinal epithelial tight junction integrity [7,12,14,48,49]. Thus, depletion of either Th17/Th22 cells and other IL-17/IL-22-producing cells such as ILCs [6] results in compromise of mucosal integrity and progression to AIDS. Therefore, therapeutic strategies aimed at preserving IL-17/IL-22 producing cells or their function during HIV infection may prevent the loss of epithelial integrity and prevent the microbial translocation which leads to immune activation and higher levels of SIV/HIV replication [7,34,41,47,50].

In conclusion, Th17 and Th22 cells display common and distinct phenotypes, and their distribution and function differs between peripheral blood and intestinal mucosal tissues in macaques. Consistent with previous reports in the colon of SIV-infected macaques [7], here we show both subsets are severely depleted during pathogenic SIV infection, and their loss correlates with disruption of the intestinal barrier and the overall loss of intestinal CD4 T cells. These findings suggest that despite their separate lineages, Th17 cells share overlapping features and functions with Th22 cells, and may synergistically play major roles in regulating homeostasis of intestinal barriers, and in HIV infection, their loss may be associated with local mucosal immune dysfunction.

## Experimental Procedures

### Animals and virus

Blood and/or tissues from a total of 93 adult Indian rhesus macaques (Macaca mulatta), which were negative for SIV, type D retrovirus, and STLV-1 infection were examined to track Th17 and/ or Th22 cells. All animals were housed at the Tulane National Primate Research Center in accordance with the Association for Assessment and Accreditation of Laboratory Animal Care International standards, and all studies were reviewed and approved by the Tulane University Institutional Animal Care and Use Committee. Of the 93 animals, 60 animals were uninfected controls, and others were chronically infected with SIVmac251 and examined in the chronic, asymptomatic stages of infection with either no overt signs of disease (chronic asymptomatic; n=33). To examine cells from intestine tissues at necropsy, macaques were euthanized for tissue collection as uninfected controls (n=60), or in chronic infection (SIV infection more than 3 months) (n=23). For some animals chronically infected with SIV, only blood was examined (n=10). Th17 cells were analyzed in blood from all 93 animals, and Th22 cells were also examined in blood from 21 uninfected, 15 chronically SIV-infected, and jejunum cells were examined from 21 uninfected and 15 chronically SIV-infected animals.

### Cell isolation and processing

Mononuclear cells from peripheral blood and intestinal tissues were isolated and processed as previously described [51]. Briefly, total peripheral blood mononuclear cells were isolated from EDTA-treated venous blood by density gradient centrifuge with Lymphocyte Separation Media (MP Biomedicals, LLC, Santa Ana, CA) as per manufacturers instruction. Tissues were collected from the jejunum within minutes of euthanasia and processed immediately for cell suspensions using enzymatic digestion as previously described [6].

### Phenotyping

Flow cytometry for surface and intracellular staining was performed using standard protocols [52]. Cells were stained with: CD3 (SP34), CD4 (L200), CCR5 (3A9), Epithelial antigen (Ber-EP4, Dako), TNF-α (MAB11), IFN-γ (B27), Granzyme B (GB11), IL-2 (MA1–17H12), IL-17 (eBio64CAP17, eBioscience), IL-21 (3A3-N2.1) and IL-22 (IL22JOP, eBioscience), Ki67 (B56), LIVE/DEAD Fixable Aqua Dead Cell Stain Kit (Invitrogen, Grand Island, NY), All antibodies and reagents were purchased from BD Biosciences Pharmingen (San Diego, CA) unless otherwise noted. Samples were resuspended in BD Stabilizing Fixative (BD Biosciences) and acquired on a FACS Calibur or LSRII flow cytometer (Becton Dickinson, San Jose, CA). Data was analyzed with Flow Jo software (Tree star, Ashland, OR).

### Ex vivo tissue culture and multi-color confocal microscopy

Fresh jejunum tissues were obtained from rhesus macaques within 30 min of necropsy and explants prepared and stimulated for detection of IL-17+ or/and IL-22+ cell subsets. Tissues were cut into 1 cm2 sections and cultured in complete RPMI medium (10% heat inactivated fetal calf serum, l-glutamine, penicillin, and streptomycin; Invitrogen) either alone (unstimulated media) or with 100ng/ml phorbol 12-myristate-13-acetate (PMA) plus 0.5mg/ml Calcium Ionophore (Stimulation medium) for 4 hours in the presence of 2µM monensin (Sigma, St.Louis, MO) to block protein transport and release. Tissues were then processed and stained as previously described [6]. In brief, Tissues were embedded and snap frozen in optimum cold temperature compound (OCT) and 7um frozen sections were stained using unconjugated primary antibodies (IL-17 and IL-22) followed by appropriate secondary antibodies conjugated to Alexa 488 (green), Alexa 568 (red) or (Molecular Probes, Eugene, OR). Formalin fixed, paraffin embedded intestinal tissues from uninfected or SIV-infected animals were stained for routine histopathology with Hematoxylin and Eosin, or for immunohistochemistry, with unconjugated primary antibodies (Desmin, D33, MS-376-S1, Fisher Scientific; Collagen, polyclonal, ab292, Abcam) and TO-PRO-3 (Life Technology) counterstaining. Confocal microscopy was performed using a Leica TCS SP2 confocal microscope equipped with three lasers (Leica Microsystems, Exton, PA). Individual optical slices representing 0.2 um and 32 to 62 optical slices were collected at 512 × 512 pixel resolution. NIH Image (version 1.62, Bethesda, MD) and Adobe Photoshop (version 7.0, San Jose, CA) were used to assign colors to the channels collected: HNPP/Fast Red (Roche, Indianapolis, IN), which fluoresces when exposed to a 568-nm wavelength laser, appears red and Alexa 488 (Molecular Probes) appears green (Figure 5A and 5B).

### Cell stimulation

Lymphocytes (106) from blood and jejunum were stimulated *in vitro* with 0.1 µM phorbol 12-myristate-13-acetate (PMA) and 0.5 µg/ml ionomycin (Sigma-Aldrich, St.Louis, MO), Cells were cultured for 4 hours in the presence of 5 µg/ml Brefeldin A (Sigma-Aldrich) then stained for cell surface markers, fixed in 2% paraformaldehyde, permeabilized in Cytofix/Cytoperm solution (BD Biosciences), and intracellularly co-stained with fluorochrome-labelled antibodies for the cytokines. For examining the effects of IL-22/IL-17A on maintenance of intestinal epithelial cells, total cell suspensions containing epithelial cells and lymphocytes isolated from the intestine were stimulated with IL-22 (10 ng/ml, BioLegend), IL-17A (10 ng/ml, BioLegend) or both and survival in vitro was compared with controls without cytokine stimulation. After 24 hours, cells were stained with Dead/lived cell staining kit and cell surface markers, and percentages of live epithelial cells were analyzed. Cells were acquired with a LSR II cytometer (Becton Dickinson). Data was analyzed with Flowjo software (Tree star, Ashland, OR).

### Statistics

Graphical presentation and statistical analysis of the data were performed using GraphPad Prism 4.0 (GraphPad Software, SanDiego, CA). Comparisons between groups were analyzed by a non-parametric Mann-Whitney U-test. P values <0.05 were considered statistically significant. Correlations between samples were determined using a non-parametric Spearman correlation analysis.
